# Translational diffusion of hydration water correlates with functional motions in folded and intrinsically disordered proteins

**DOI:** 10.1038/ncomms7490

**Published:** 2015-03-16

**Authors:** Giorgio Schirò, Yann Fichou, Francois-Xavier Gallat, Kathleen Wood, Frank Gabel, Martine Moulin, Michael Härtlein, Matthias Heyden, Jacques-Philippe Colletier, Andrea Orecchini, Alessandro Paciaroni, Joachim Wuttke, Douglas J. Tobias, Martin Weik

**Affiliations:** 1 IBS, Univ. Grenoble Alpes, IBS, F-38044 Grenoble, France; 2 CNRS, IBS, 71 avenue des Martyrs, F-38044 Grenoble, France; 3 CEA, IBS, F-38044 Grenoble, France; 4 Department of Chemistry, University of California, Irvine, California 92697-2025, USA; 5 Institut Laue-Langevin, 71 avenue des Martyrs, 38000 Grenoble, France; 6 Australian Nuclear Science and Technology Organisation Bragg Institute, Menai, New Illawarra Road, Lucas Heights, NSW 2234, Australia; 7 ILL-EMBL Deuteration Laboratory, Partnership for Structural Biology, 38044 Grenoble, France; 8 Max-Planck-Institut für Kohlenforschung, 45470 Mülheim an der Ruhr, Germany; 9 Dipartimento di Fisica e Geologia, Università di Perugia, Via Pascoli, 06123 Perugia, Italy; 10 Forschungszentrum Jülich, JCNS at MLZ, Lichtenbergstrasse 1, 85747 Garching, Germany

## Abstract

Hydration water is the natural matrix of biological macromolecules and is essential for their activity in cells. The coupling between water and protein dynamics has been intensively studied, yet it remains controversial. Here we combine protein perdeuteration, neutron scattering and molecular dynamics simulations to explore the nature of hydration water motions at temperatures between 200 and 300 K, across the so-called protein dynamical transition, in the intrinsically disordered human protein tau and the globular maltose binding protein. Quasi-elastic broadening is fitted with a model of translating, rotating and immobile water molecules. In both experiment and simulation, the translational component markedly increases at the protein dynamical transition (around 240 K), regardless of whether the protein is intrinsically disordered or folded. Thus, we generalize the notion that the translational diffusion of water molecules on a protein surface promotes the large-amplitude motions of proteins that are required for their biological activity.

## Abstract (web summary)

Hydration water plasticizes protein structures and is essential for their biological functions, such as enzymatic catalysis. Here, the authors use neutron scattering and molecular dynamics simulations to study hydration water at the dynamical transition of folded and disordered proteins.

## Graphical abstract

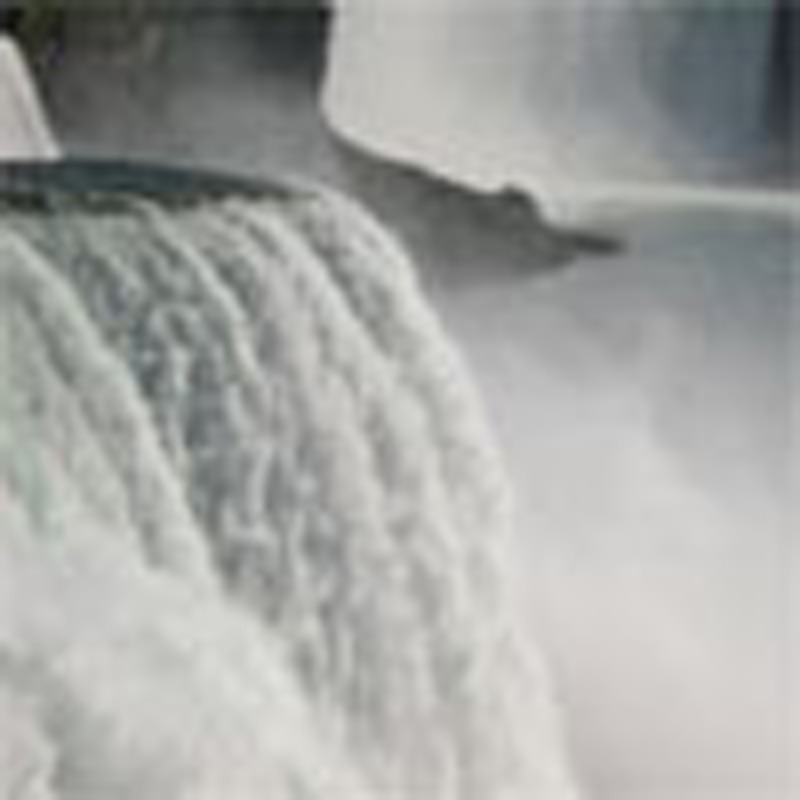

Hydration water surrounds biological macromolecules such as soluble proteins. Rather than being a mere bystander, hydration water actively partakes in macromolecular activity such as protein–protein and protein–DNA recognition, protein allostery, electron and proton transfer, and enzyme reactions^1^. The first hydration layer on the protein surface is of particular importance for biological activity that is lost in most completely dry proteins^2^. A protein together with its first hydration layer thus forms the biologically active entity. Protein and water molecules are connected by an extended hydrogen-bonded network, the fluctuations of which lead to breakage and formation of water–protein hydrogen bonds (HBs) that eventually allow for functionally important protein motions at physiological temperatures^3^. The coupling between water and protein dynamics has been, and remains, a matter of extensive debate. The relation has been compared with the one between a master (water) and a slave (protein dynamics)^4^, yet a mutual give-and-take analogy probably best accounts for the experimentally observed differential influence of different classes of biological macromolecules (globular, membrane and intrinsically disordered proteins (IDPs)^5^, DNA and RNA^6^) on the dynamics of their hydration water. The complex ensemble of water–protein motions can be teased apart by extending experiments and simulations down to cryo-temperatures; hydrated proteins undergo a transition to large-amplitude anharmonic motions on the picosecond timescale as the temperature is increased above ~240 K. The origin of this transition, known as the protein dynamical transition^7^, is controversially debated in the biophysical community^8^,^9^,^10^,^11^,^12^,^13^,^14^. A transition is also apparent in the picosecond motions of hydration water at a similar temperature^15^. The hydration water transition has been suggested to either be of kinetic origin and the manifestation of a glass transition at around 180 K^16^,^17^,^18^ or due to a thermodynamic transition between a high- and a low-density form of water^19^. Despite the various controversies, there is a general agreement about the primary role played by hydration water dynamics in protein dynamics and function, as decreasing the hydration level significantly below monolayer coverage effects in suppressing both the dynamical transition^20^,^21^ and the biological activity^2^,^22^ of a protein. Molecular dynamics (MD) simulations have suggested that the onset of water translational diffusion on the protein surface correlates with the protein dynamical transition, so that water and protein motions emerge in parallel on the same timescale^23^,^24^. Yet, experimental evidence for such a correlation has remained elusive and its universal validity for different protein classes, including IDPs, unexplored.

In contrast to folded proteins, IDPs lack a single, well-defined three-dimensional structure and are best described by an ensemble of rapidly interconverting conformations^25^. The unfolded nature of IDPs originates from a large content of hydrophilic amino acid residues compared with hydrophobic ones, preventing the hydrophobic collapse that would lead to a folded globular protein^26^. IDPs fulfill specific roles in biological cells, where they are mainly involved in signalling and regulatory processes^27^,^28^,^29^, and in many cases fold on binding to their target partner^30^. About 30% of eukaryotic proteins are thought to be either partially or fully disordered^31^. IDPs are more flexible than folded proteins, as shown, for example, by neutron^5^,^32^ and NMR spectroscopies^33^. Yet, similar to folded proteins IDPs undergo a dynamical transition^5^ that is suppressed if hydration water is absent. Owing to their unfolded nature, IDPs are characterized by a solvent accessible surface exceeding several times the one of folded globular proteins. One might thus conjecture that the interaction with hydration water is different for IDPs than for globular proteins.

Neutron scattering is a valuable tool to study the nano- to picosecond (ns–ps) dynamics of biological macromolecules and of hydration water^34^. The incoherent scattering cross-section of a hydrogen atom exceeds by two orders of magnitude the ones of all other atoms in a protein, including its isotope deuterium, and thus dominates the neutron scattering signal in non-crystalline samples. Therefore, fully deuterating a protein (perdeuteration) is an elegant way to focus on hydration water dynamics by minimizing the protein’s contribution to the incoherent scattering signal. Yet, perdeuterating a protein in the large quantities needed for energy-resolved incoherent neutron-scattering experiments (100–200 mg) is time consuming, costly and has been carried out for only a handful of proteins so far, including C-phycocyanin^35^, purple membrane^36^, maltose binding protein (MBP)^15^, myoglobin^37^, tau^5^ and green fluorescent protein^18^. For powders of perdeuterated proteins hydrated at a level of 0.4 g H_2_O per gram protein, at least 70% of the incoherent neutron scattering signal is from hydration water that corresponds roughly to monolayer coverage of the protein surface^36^. The remaining ~30% of the signal originates essentially from exchanged hydrogen atoms of the protein.

All-atom MD simulations are complementary to neutron-scattering experiments. MD simulations routinely cover the same timescale (ns–ps) of scattering experiments and it is straightforward to compute neutron-scattering spectra from MD trajectories. Comparison of MD and neutron scattering data is useful for validating the simulations and, once validated, MD simulations can scrutinize models used to interpret the scattering data. MD simulations have been widely used to characterize the vibrational and diffusional properties of protein hydration water^15^,^23^,^24^,^38^,^39^,^40^,^41^,^42^,^43^.

Here we studied hydration water dynamics on the surface of the folded MBP and of the intrinsically disordered human protein tau that is, similar to many IDPs, involved in a neurodegenerative disease, *viz*. in Alzheimer’s disease^44^. A combination of protein perdeuteration, quasi-elastic neutron scattering (QENS) and MD simulations provided evidence for an activation of translational water motions on tau and MBP at the temperature where the proteins undergo a dynamical transition. We establish a general connection between the translational diffusion of water molecules on a protein surface and the promotion of the large amplitude motions of proteins required for their biological activity. This connection is thus independent of the protein-folding state.

## Results

### Neutron scattering reveals a change in water dynamics at 240 K

**Figure 1:**
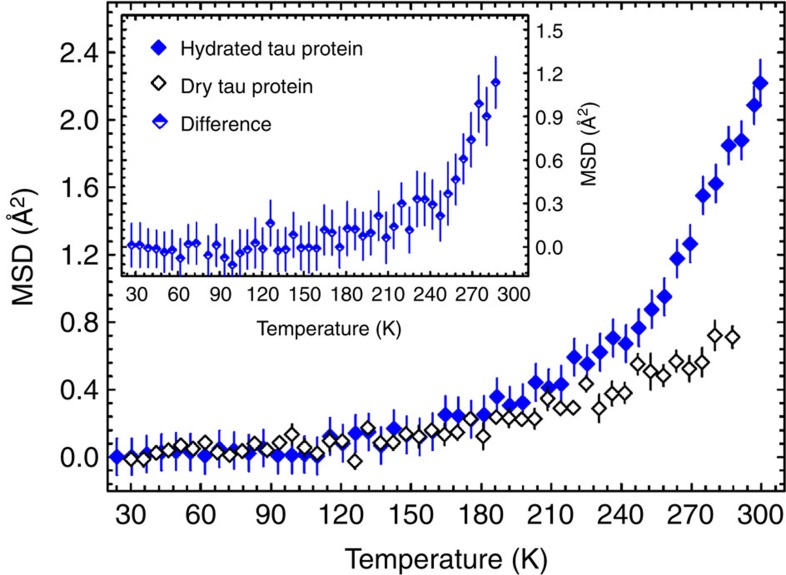
Dynamical transition in hydrated proteins. MSDs of the IDP tau^5^ in a hydrated (full blue diamonds) and a dry (open black diamonds) state, measured by elastic incoherent neutron scattering on the backscattering spectrometer IN16 (0.9 μeV resolution, ILL, Grenoble)^69^. Inset: the MSD difference between the hydrated and the dry protein highlights an onset of large-amplitude protein motions at around 240 K. Protein samples were not deuterated and were hydrated in D_2_O. Experimental error bars are indicated as vertical lines.

The htau40 protein (the longest and most commonly found isoform of human tau, referred to as tau throughout the manuscript) and the MBP are of similar molecular mass (corresponding to 441 and 380 amino acids, respectively), yet they belong to different protein families; the former is an IDP, whereas the latter is fully folded. Hydrated powders of both proteins have been prepared and characterized earlier^5^. Elastic incoherent neutron-scattering experiments on D_2_O-hydrated, non-deuterated tau^5^ and non-deuterated MBP^15^ samples have indicated that a protein dynamical transition (Fig. 1) takes place at a similar temperature in both proteins. This temperature can be estimated by subtracting the mean-squared displacements (MSDs) of hydrated tau from those of dry tau (see inset in Fig. 1) and calculating the second derivative that shows a sharp peak close to 240 K (Supplementary Fig. 1).

**Figure 2:**
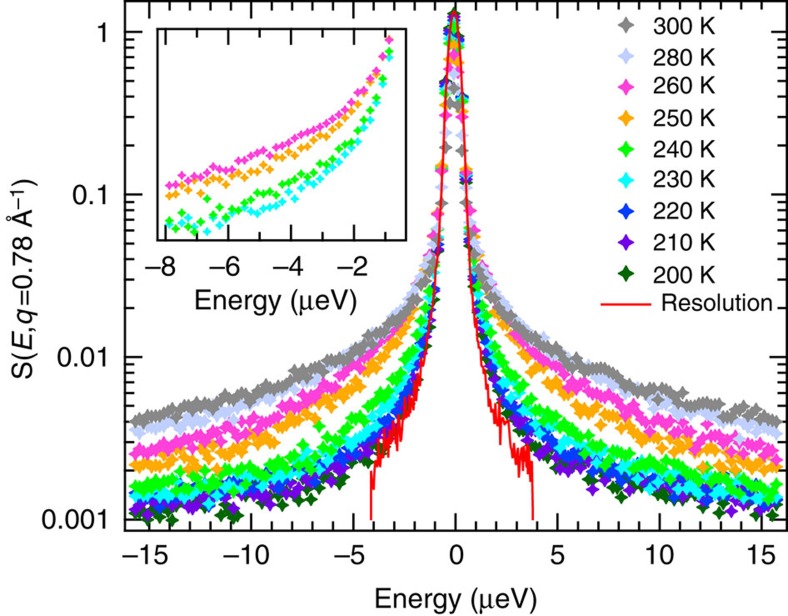
Neutron spectra reveal a change in hydration water dynamics at 240 K. QENS spectra of D-tau-H_2_O at different temperatures and at *q*=0.78 Å^−1^. The spectrum in red corresponds to the experimental resolution function, obtained by a measurement of the same sample at 20 K, and was truncated below and above −4 and 4 μeV. Inset: zoom into the quasi-elastic spectra between −8 and −0.6 μeV for 230, 240, 250 and 260 K, highlighting the change at 240 K.

In the present study, QENS measurements were carried out on H_2_O-hydrated, perdeuterated tau (denoted D-tau-H_2_O) and H_2_O-hydrated, perdeuterated MBP (D-MBP-H_2_O) in an ample temperature range across the protein dynamical transition, that is, between 200 and 300 K, to monitor hydration water dynamics. The data were recorded on the backscattering spectrometer SPHERES^45^ (Jülich Centre for Neutron Science at MLZ, Garching, Germany) with an energy resolution of Δ*E*=0.7 μeV (full width at half maximum) that allows monitoring of motions in the ns–ps timescale. The neutron-scattering signal shows quasi-elastic broadening at temperatures above 200 K, indicating the presence of ns–ps motions in the hydration shell. A representative description of the observed temperature dependence is given in Fig. 2, where the spectra of tau hydration water are reported at all measured temperatures, normalized for the spectral area measured at 20 K and at the scattering wavenumber *q*=0.78 Å^−1^ (*q*=*λ*^−1^4*π*sin(*θ*/2), where *λ* is the incident neutron wavelength and *θ* the scattering angle). An abrupt change in the spectral lineshape occurs between 240 and 250 K (Fig. 2; an analogous behaviour is revealed in MBP and at all the measured *q*-values (not shown)). Such a change reveals that a modification in the dynamics of hydration water takes place on the ns–ps timescale in this temperature region, which corresponds to the protein dynamical transition temperature revealed by elastic data on D_2_O-hydrated samples (Fig. 1).

### Data analysis reveals an onset of water translation at 240 K

**Figure 3:**
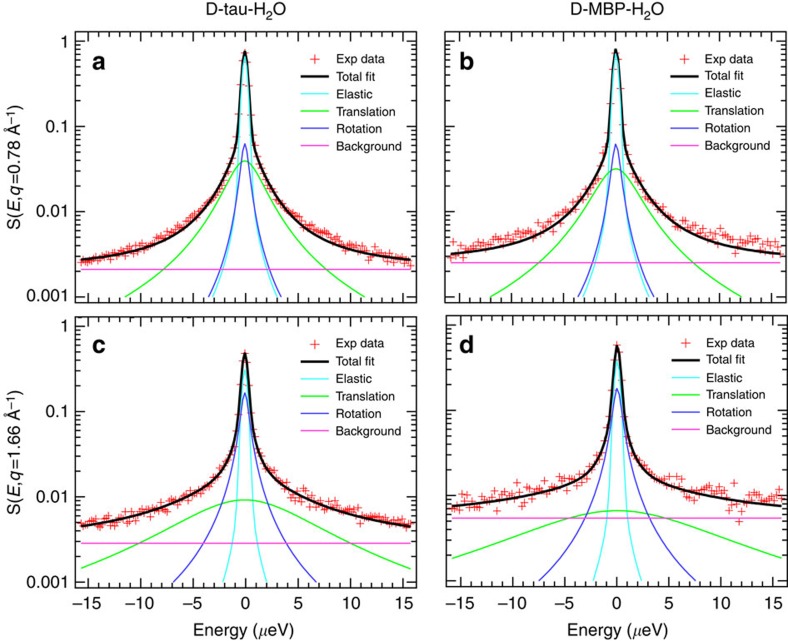
Neutron spectra and fits. QENS spectra of D-tau-H_2_O (**a**,**c**) and D-MBP-H_2_O (**b**,**d**) at 260 K and for *q*=0.78 Å^−1^ (**a**,**b**) and *q*=1.66 Å^−1^ (**c**,**d**). The continuous lines represent the fitting curves with a model in which water molecules either translate, rotate or remain immobile.

Water diffusional motions can be described as a translation of the centre-of-mass and a rotation around the centre-of-mass. Here we employ a model for analysing QENS data in which water molecules either translate, rotate or remain immobile (see Methods section). Furthermore, we adopted a global fitting approach, in which the difference between experimental data and the fitting function is minimized in the two-dimensional space defined by energy transfer (*E*) and scattering wavenumber (*q*). This procedure minimizes the number of free parameters and allows the identification of contributions to the QENS spectrum arising from confined motions (such as rotational motions). Indeed, as these contributions have a typical length scale (for example, the radius of a rotational motion), their intensity is *q*-dependent and can vary significantly in the accessible *q*-range. In Fig. 3, we show typical results of the fitting procedure at two different *q*-values, illustrating the excellent agreement of the model with the experimental data (fitting results at other temperatures and *q*-values are reported in Supplementary Fig. 2). Data shown in the upper panels of Fig. 3 highlight the importance of fitting the spectra by simultaneously taking into account their *q*-dependence. Indeed, the intensity of the rotational contribution cannot be distinguished from the immobile contribution (contained in the elastic peak in Fig. 3) when analysing single spectra at low *q*-values.

**Figure 4:**
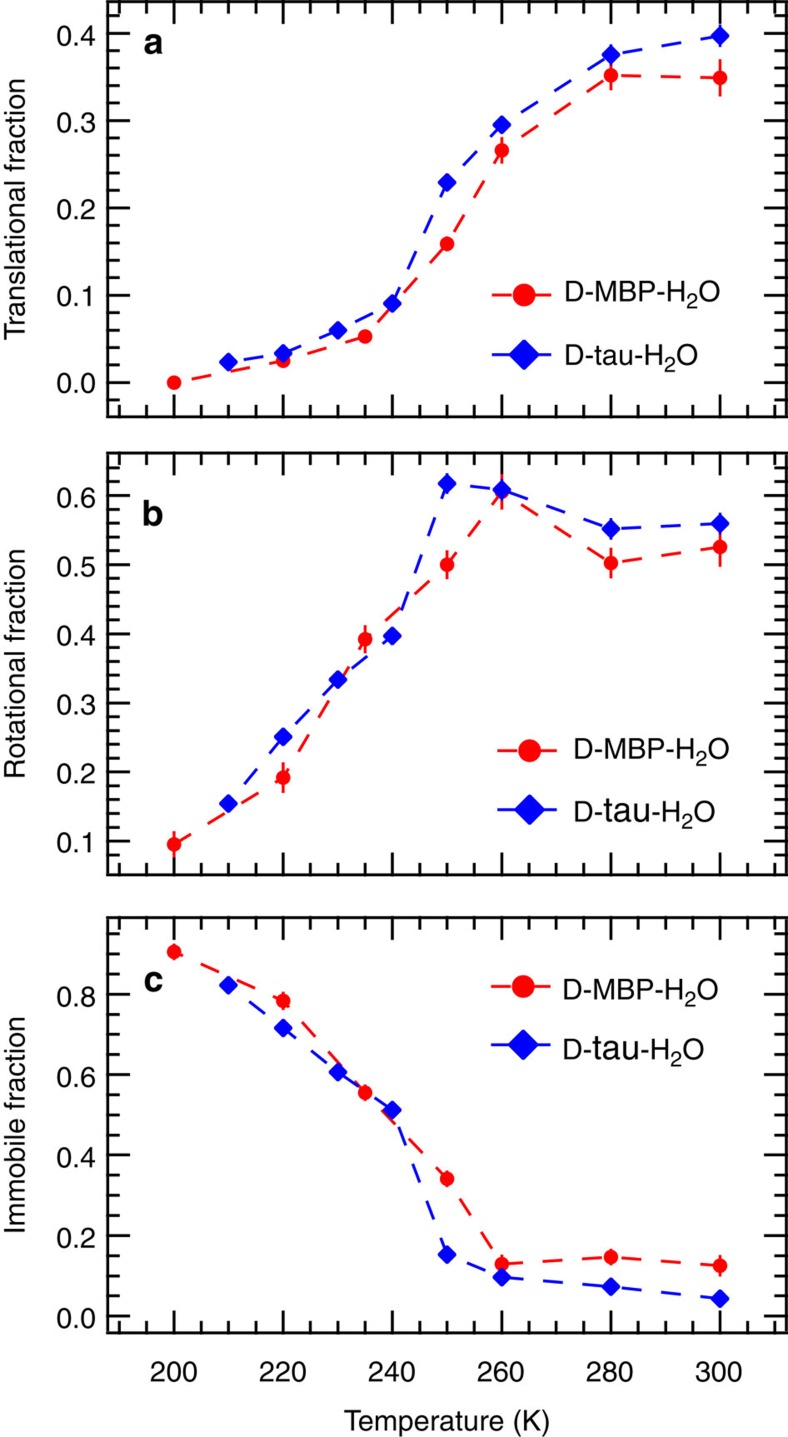
Different populations of water molecules as a function of temperature. Fractions of different dynamic contributions to the quasi-elastic spectra as a function of temperature: centre-of-mass translation of water molecules (**a**), rotation of water molecules around their centre-of-mass (**b**) and water molecules not moving in the dynamic window investigated (**c**). Dashed lines are guides to the eye. Red circles, MBP; blue diamond, tau.

From the global fit analysis, we can extract for each temperature point fractions of the total scattering intensity originating from water molecules that undergo translational or rotational diffusion, or that remain immobile. These fractions are displayed in Fig. 4 for both tau and MBP hydration water as a function of temperature. For both tau and MBP, the translational fraction increases steeply above 240 K (Fig. 4a). The rotational fraction has emerged at 200 K and increases up to 250 K, where it reaches a plateau (Fig. 4b). Below 200 K, nearly all water molecules are thus immobile (Fig. 4c) on the timescale determined by the SPHERES spectrometer (motions faster than 2 ns). Additional QENS experiments were carried out at various temperatures on natural-abundance tau and MBP powders, hydrated in D_2_O (results to be published in detail elsewhere), and a contribution from the resulting protein dynamics included in equation (1). The resulting temperature dependence of translational, rotational and immobile fractions of water molecules was qualitatively similar to the ones shown in Fig. 4. The quasi-elastic data (Fig. 2) were collected in the energy range ±15.8 μeV (see Methods section). To uncover a possible effect of the limited energy range on data analysis, we also collected a set of QENS spectra of D-tau-H_2_O at four selected temperatures (240, 260, 280 and 300 K) in the full-energy range available on SPHERES (±30.9 μeV). The extracted translational, rotational and immobile fractions are almost identical to the ones displayed in Fig. 4 (see Supplementary Fig. 3). The fitting model (equation (1)) also contains a background (that is, *k*(*q*)) originating from an instrumental background and fast motions outside the dynamic range of the spectrometer. *k*(*q*) appears to display a temperature dependence with a transition at 240 K for all *q*-values (Supplementary Fig. 4), yet the rate of increase above 240 K is *q*-dependent. A *q*-dependent background with a transition at 240 K is compatible with a fast translational water component (detected by SPHERES as a flat energy-independent contribution; see Supplementary Fig. 5 and Supplementary Information for a complete discussion) that shows a similar temperature dependence than the slower component shown in Fig. 4a. The other dynamical parameters (that is, rotational correlation rate and translational diffusion coefficient) reveal a perturbation of protein hydration water with respect to bulk water as already reported in the literature (see Supplementary Fig. 6).

### MD simulations confirm the onset of water translation

**Figure 5:**
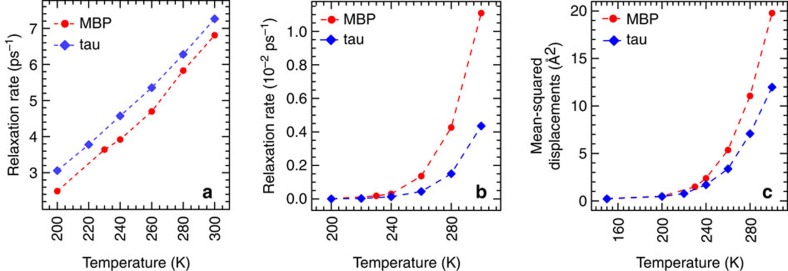
Analysis of MD simulations of hydration water dynamics on tau and MBP surfaces. (**a**) Continuous HB relaxation rates (1/*τ*_HBC_) as a function of temperature. (**b**) Intermittent HB relaxation rates (1/*τ*_HBI_) as a function of temperature. (**c**) MSDs of hydration water oxygen atoms at 100 ps as a function of temperature. Error bars on the relaxation rates and MSDs, estimated by computing the respective quantities separately over the two halves of the trajectory segments used for the analysis, are smaller than the plotting symbols.

To complement the QENS results and to confirm their connection with previous computational work^23^, we carried out MD simulations on tau and MBP. Models of protein powders hydrated at 0.4 g H_2_O per gram protein were prepared and the simulations were carried out at constant pressure and temperature for ~50 ns and analysed. The agreement between simulation and experiment of the temperature evolution of the water MSD was found to be excellent for both MBP and tau (Supplementary Fig. 7), thus validating our simulations and justifying further *in silico* analyis of water dynamics. We evaluated the temperature dependence of two types of protein–water hydrogen bond (HB) lifetimes computed from the simulations: the continuous HB relaxation time (also known as the fast HB relaxation time), *τ*_HBC_, and the intermittent HB relaxation time (also referred as the slow HB network relaxation time), *τ*_HBI_. *τ*_HBC_ is the average time before a protein–water HB breaks, while *τ*_HBI_ represents the timescale for relaxation of the protein–water HB network^46^. Relaxation rates, defined as 1/*τ*, are plotted as a function of temperature in Fig. 5a,b. The continuous HB relaxation rates (Fig. 5a) show a smooth variation over the entire temperature range for both protein powders. In contrast, the intermittent HB relaxation rate (fig. 5b) exhibits a sharp increase at around 240–260 K. Furthermore, we computed for both protein powders the MSD of the hydration water oxygen atoms, assessing thereby the translational dynamics of water molecules. The MSD, plotted at 100 ps as a function of temperature in Fig. 5c, also show a sharp increase at around 240 K, signalling the onset of water translational diffusion.

## Discussion

We addressed the relation between hydration water motions and the dynamics of both an IDP (human htau40) and a folded protein (MBP) by combining protein perdeuteration, neutron scattering and MD simulations. QENS of perdeuterated proteins hydrated in H_2_O, which enables the isolation of hydration water motions, was measured at several temperatures between 200 and 300 K. The quasi-elastic signal broadens abruptly at 240 K, indicating that hydration water motions become activated on the nanosecond timescale at a similar temperature where the underlying protein undergoes a dynamical transition that corresponds to the onset of anharmonic motions. Fitting the broadening with a model in which water molecules either translate, rotate or remain immobile provided evidence for an onset of water translational motions at the protein dynamical transition of both MBP and tau. The freezing of all water-diffusion degrees of freedom around 200 K on the nanosecond timescale is in agreement with the presence of a second order-like glass transition of hydration water at a lower temperature on a longer timescale (100 s), as revealed by dielectric spectroscopy and calorimetry^47^.

A previous MD simulation study of the globular protein RNase A established a correlation between the relaxation of the protein–water HB network and the onset of anharmonic motions that occurs at the protein dynamical transition^23^. In the present study, MD simulations of tau and MBP at various temperatures below and above the dynamical transition showed that the lifetimes (*τ*_HBI_) of the protein–water HB networks abruptly decrease at the dynamical transition, concomitant with the onset of translational water diffusion. The observation of this coincidence for two globular proteins (RNase A^23^ and MBP), as well as for an entirely disordered protein (tau), suggests a universality of the correlation between an abrupt increase in the HB network relaxation rate and the dynamical transition.

The hydration water properties of IDPs are thought to have specific biological relevance, such as ensuring protein activity in desiccated cells^48^, but they remain poorly understood. Solid-state NMR revealed larger activation energies for IDP than for globular protein hydration water motions and indicated that IDPs bind more hydration water than globular proteins^49^. ^17^O magnetic relaxation dispersion experiments suggested that IDPs with extended conformations display weaker dynamical perturbations of their hydration water than folded proteins^50^. Elastic neutron scattering has provided evidence of smaller mean-square displacements for the hydration water of an IDP compared with a globular protein^5^. Despite these differences that might be due to the conformational heterogeneity and the amino acid composition of IDPs that differ from those of folded proteins, our results show that water translational motions couple to large-amplitude protein motions in both globular and IDPs. Among different classes of soluble proteins, the coupling between hydration water and protein motions thus seems to be of similar nature.

Concerning the water diffusive model used here, we underline that a much better fitting quality is obtained with a function where the translational and rotational terms are added (see equation (1)) rather than convoluted (*viz. χ*^2^ is at least 20% higher on average, but even 50% higher at temperatures above 250 K; see Supplementary Fig. 8). This indicates the presence of two populations of water molecules with different dynamical properties within the dynamical window probed by the SPHERES neutron spectrometer (ns–ps motions). The existence of two populations is supported by water displacement distributions calculated from the MD simulations (Supplementary Fig. 9). The rotational term accounts mainly for water molecules strongly interacting with the protein surface and/or inside protein docking sites, while the translational term accounts for less hindered water molecules whose rotational motion is faster than the time window accessible by SPHERES. This finding is in agreement with the previously observed heterogeneity of water motions in the first hydration shell^51^ and is in agreement with the experimental evidence that a certain protein hydration level (typically *h*>0.15 g water/g protein) is needed for the dynamical transition to take place: at lower hydration levels, water molecules are probably so tightly bound to the protein surface that only rotational motions are energetically accessible, while translational motions are not allowed and, as a consequence, the full set of protein motions required for the dynamical transition to take place can not be activated. Extending the present study to lower hydration levels would further clarify this point.

In summary, the combination of simulation and experimental data reported herein confirms the notion that the protein dynamical transition is connected to the onset of water translational diffusion via relaxation of the protein–water HB network. A key aspect of our approach was a line width analysis of QENS data that yielded insight into the nature of water motions not provided by MSDs deduced from elastic scattering. The similarity of the results obtained for the globular protein MBP and the IDP tau suggests, unexpectedly, that this connection does not depend on the degree of order in a protein structure, and is a generic feature of the dynamics of soluble proteins. The protein dynamical transition has been shown to be correlated with the onset of biological activity^2^,^22^,^52^. Our data thus suggest that the ability of hydration water to undergo translational motions on a protein surface is a necessary requirement for protein activity. Extrapolated to the context of a biological cell, we conjecture that macromolecular activity might be modulated via a modification in the translational diffusion properties of hydration water. Such a modification can, for example, be brought about by geometric confinement, macromolecular crowding or the presence of small solutes such as sugar molecules.

## Methods

### Sample preparation for neutron-scattering experiments

We used the same perdeuterated htau40 (D-tau-H_2_O; ref. 5) and perdeuterated MBP (D-MBP-H_2_O)^15^ samples as employed in our previous studies. Details about sample preparation and characterization, and chemicals used can be found in Gallat *et al*.^5^ (tau) and Wood *et al*.^15^ (MBP). Briefly, 204 mg of perdeuterated htau40 and 221 mg of perdeuterated MBP were hydrated to a level of 0.38 g H_2_O/g D-tau (D-tau-H_2_O sample; corresponding to 1,040 water molecules per tau molecule) and 0.37 g H_2_O/g D-MBP (D-MBP-H_2_O sample; corresponding to 910 water molecules per MBP molecule), respectively, in a 4 × 3 cm^2^ flat aluminum sample holder. If one assumes that all exchangeable deuterons in the D-tau (D-MBP) protein, that is, 24% (22%) of all deuterons in the protein, exchange, 71% (72%) of the total incoherent scattering cross-section originates from hydration water (H_2_O) and 29% (28%) from the protein.

### Quasi-elastic incoherent neutron-scattering experiments

QENS data were recorded on the backscattering spectrometer SPHERES (Jülich Centre for Neutron Science at the Heinz Maier-Leibnitz-Zentrum Garching, Garching, Germany) with a scattering wavenumber range *q*=0.46–1.66 A^−1^, an energy resolution Δ*E*=0.7 μeV (full width at half maximum)^45^ and in the energy range *E*=±15.8 μeV, corresponding to a ns–ps time window. The samples were inserted at room temperature into a Janis cryostat at 135° with respect to the incoming neutron beam. QENS spectra were collected at several temperatures in the following sequence: 20, 200, 210, 220, 230, 240, 300, 280, 260 and 250 K for D-tau-H_2_O and 20, 250, 200, 220, 235, 260, 280 and 300 K for D-MBP-H_2_O. The total measuring time per temperature was 10 h (20–220 K) or 9 h (230–300 K) for D-tau-H_2_O and 12 h for D-MBP-H_2_O. Detector efficiencies were corrected with a measurement of the scattering intensities from the samples at 20 K. Data points were binned in 0.04 μeV channels. Spectra at some selected temperatures were also collected in the full ±30.9 μeV energy range accessible on SPHERES, to check possible effects of the accessible energy window on the results of data analysis (see Supplementary Fig. 3).

### Neutron data analysis

QENS data were analysed using a rotation–translation model^53^ for hydration water dynamics with four components: a translational term, taking into account the centre-of-mass motion of water molecules; a rotational term, accounting for motions around the centre-of-mass; a term for the ‘immobile’ water molecules (that is, the waters characterized by motions too slow to be detected in the dynamic range probed by the neutron spectrometer); and a flat energy-independent term for the instrumental background and for motions faster than the time window probed by the spectrometer. All the energy-dependent terms were convoluted with the instrumental resolution function, which was obtained by measuring the spectra of the same samples at 20 K, where the incoherent scattering signal is almost exclusively of elastic nature. Both translational and rotational terms have a Lorentzian-like dependence on energy transfer, thus making them difficult to distinguish when analysing a single spectrum at a given *q*-value. The main difference between the translational and rotational terms lies in the *q*-dependence of their shape parameters: the first one has a *q*-dependent width and a *q*-independent intensity, while the opposite holds for the second one. These characteristics allow us to distinguish and assign the two different dynamical contributions to the QENS spectrum when using a global fitting approach in the *q–E* space, similar to the one adopted here.

The model for the scattering function *S*(*q*,*E*) has the following analytical form:


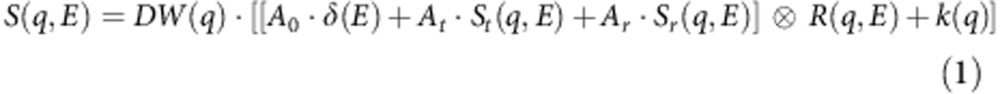


*DW*(*q*)=exp(−*q*^2^‹*u*^2^›) is the Debye–Waller factor due to the vibrational motions. The intensity of the elastic term (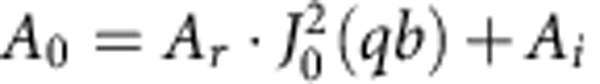, where *J*_*0*_ is the 0th order spherical Bessel function) contains a *q*-dependent term from the rotational diffusion model and a *q*-independent term *A*_*i*_ that takes into account hydrogen atoms not moving on the timescale set by the resolution function. 
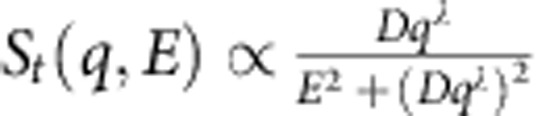
 is the contribution of translational motions (for *λ*=2 the standard Brownian diffusion is recovered, while *λ*<2 indicates a sub-diffusive character, often found in polymers, glass-forming and supercooled liquids)^54^. 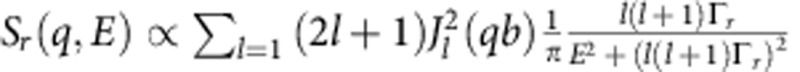 is the quasi-elastic broadening originating from rotational motions^55^. *J_l_(qb)* are the spherical Bessel functions, while *b* is the radius of the hydrogen rotation around the centre-of-mass of a water molecule, which was set to 0.98 Å, that is, the H–O distance (the centre-of-mass position essentially coincides with the oxygen atom position). The first five terms of the series, accounting for more than 99% of the rotational term in the explored *q*-range, were taken into account. *k(q)* is an energy-independent background that accounts for all the contributions faster than the timescale detected in the SPHERES energy range, as well as the instrumental background. The free parameters in this model are then: ‹*u*^*2*^›, *A*_*i*_, *A*_*t*_, *D*_*t*_, *λ*, *A*_*r*_, Γ_*r*_ and *k(q)* for a complete data set at a given temperature. The fitting procedure consists in minimizing the function 
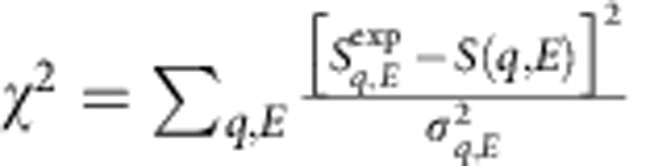
, where the summation is over all the (*q*,*E*) values, *S*^exp^ are the experimental points and *σ* the experimental errors associated with the experimental points. To carry out the fitting, a Fortran code was written, based on the Minuit minimization routine (http://lcgapp.cern.ch/project/cls/work-packages/mathlibs/minuit/index.html), released by the CERN computing group.

### MD simulations

The MD simulations were performed using the NAMD programme^56^ with the CHARMM27 force field^57^,^58^ for the protein and the SPC/E^59^ model of water. The SPC/E model has been shown to more accurately reproduce neutron-scattering data on protein hydration water than the TIP3P water model that is typically used in conjunction with the CHARMM protein force field^43^. The simulations were carried out at constant temperature using Langevin dynamics and a constant pressure of 1 atm using the Nosé–Hoover–Langevin piston algorithm with anisotropic cell fluctuations^60^,^61^. The equations of motion were integrated using the Verlet-I/r-RESPA multiple-time step algorithm^62^,^63^ with time steps of 4 fs for the long-range non-bonded forces, 2 fs for the short-range non-bonded forces and 2 fs for the bonded intramolecular forces. The SHAKE algorithm^64^ was used to constrain the lengths of all bonds to H atoms. Electrostatic interactions were computed using the smooth particle-mesh Ewald sum^65^, and the van der Waals interactions and the real-space part of the Ewald sum were smoothly switched to zero over the range 10–11 Å for the MBP powder and 10–12 Å for the tau powder.

The MBP powder model was prepared as described earlier^15^. Briefly, the powder consists of four molecules of MBP (PDB entry 1JW4) and 3,460 water molecules, corresponding closely to the hydration level of the samples used in the neutron experiments. The tau powder is composed of ten tau molecules and was prepared as follows. Out of an ensemble of 200 tau conformations determined by NMR and small angle X-ray scattering experiments^66^, we randomly selected a sub-ensemble of ten conformations, for which the average radius of gyration matched the experimental value measured of tau in solution (62 Å)^5^. Those ten conformations were placed in the same simulation box before adding 10,105 water molecules (corresponding to the experimental hydration level of 0.4 g H_2_O per g protein) spread over the first hydration shell of each protein. Snapshots from the simulations of both the tau and MBP powder models are shown in Supplementary Fig. 10. We then started the simulation at constant temperature and pressure, so that the simulation box collapsed. After the box dimensions had stabilized, we set the temperature to 500 K for 1 ns. The temperature was then reduced to 300 K, the simulation was prolonged for an additional 2 ns and the final configuration was used as a starting point for the simulations at a series of temperatures. The two powder models were equilibrated for about 30 ns at 20, 150, 200, 230, 240, 260, 280 and 300 K for MBP and 20, 150, 200, 220, 240, 260, 280 and 300 K for tau, followed by 14 ns of simulations at each temperature during which configurations were saved at 1-ps intervals for subsequent analysis. For the calculations of the protein–water HB lifetimes (*τ*_HBC_, defined below), the simulations were continued for another 400 ps, during which configurations were saved at 8-fs intervals.

The water dynamics in our powder models were validated by comparing the computed and experimental water MSDs as a function of temperature, as shown in Supplementary Fig. 7. The temperature dependence of the MSD from the simulations agrees well with the experimental data, although the absolute values are different for reasons discussed in the Supplementary Information.

Two measures of protein–water HB relaxation rates were considered, to discriminate between fast (~ps) HB formation/breakage (characterized by the ‘continuous’ HB relaxation time *τ*_HBC_) due to water rotational/librational motion and the slower (>10 ps) relaxation of the protein–water HB network (characterized by the ‘intermittent’ HB relaxation time *τ*_HBI_) due to diffusion of water on the protein surface or exchange of water molecules between hydration shells. *τ*_HBC_ is defined by the decay of the time correlation function *S*_HB_*(t)*=*‹h(0)H(t)›/‹h›* (ref. 67) and *τ*_HBI_ by the decay of *C*_HB_*(t)=‹h(0)h(t)›/‹h›* (ref. 68), where *h*(*t*) is equal to 1 if a given donor–acceptor (D–A) pair is hydrogen bonded at time *t* and zero otherwise, *h(t)* is equal to 1 if the D–A HB remains intact continuously from time 0 to time *t* and zero otherwise, and the angular brackets denote an average over all D–A pairs and time origins. The relaxation times *τ*_HBC_ and *τ*_HBI_ were defined as the times at which *S*_HB_*(t)* and *C*_HB_*(t)* decay, respectively, to 1/*e*. When the *C*_HB_*(t)* did not decay to 1/*e* within 4 ns, they were fitted with a stretched exponential and extrapolated to 1/*e*.

For the calculation of the correlation functions *C*_HB_*(t)* and *S*_HB_*(t)*, a D–A pair was considered to be hydrogen bonded when the distance D–A was <3.5 Å and the D–H–A angle was greater than 150°. Although the values of the HB lifetimes (*τ*_HBC_) at a given temperature depend on the details of the criterion used to define HBs, previous work on bulk water suggests that the definition is not expected to affect the qualitative temperature dependence of the lifetimes^46^.

## Author contributions

M.W. proposed experiments. F.-X.G., J.-P.C., M.M. and M. Härtlein prepared samples. F.-X.G., M.W. and J.W. performed neutron experiments. Y.F., M. Heyden and D.J.T. carried out and analysed the MD simulations. G.S. analysed neutron data with input from J.W., F.-X.G., A.O., A.P., K.W., F.G. and M.W. Y.F., G.S., D.J.T. and M.W. wrote the manuscript, with input from all authors.

## Additional information

**How to cite this article**: Schirò, G. *et al*. Translational diffusion of hydration water correlates with functional motions in folded and intrinsically disordered proteins. *Nat. Commun.* 6:6490 doi: 10.1038/ncomms7490 (2015).

## Supplementary Material

Supplementary Information Supplementary Figures 1-10, Supplementary Discussion and Supplementary References
