# Supplementary material for: Translational diffusion of hydration water correlates with functional motions in folded and intrinsically disordered proteins
Source: Nat Commun. 2015 Mar 16;6:6490. doi: 10.1038/ncomms7490 (PMC4382692; doi:10.1038/ncomms7490)
Supplement: Supplementary Information — Supplementary Figures 1-10, Supplementary Discussion and Supplementary References [file ncomms7490-s1.pdf]

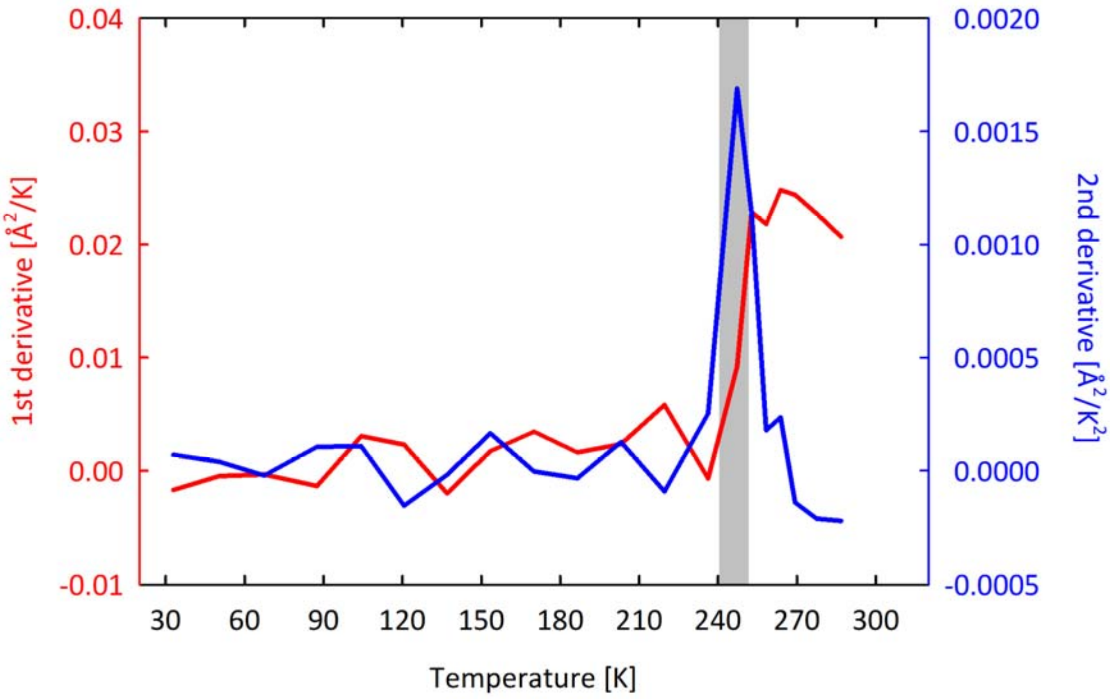

Supplementary Figure 1: **First (red curve, left axis) and second (blue, right axis) derivative of the MSD difference between the hydrated and the dry tau protein reported in the inset of Figure 1.** The presence of a step and a peak in the first and the second derivative, respectively, between 240 and 250 K (indicated by the grey area) defines the temperature region where the dynamical transition occurs.

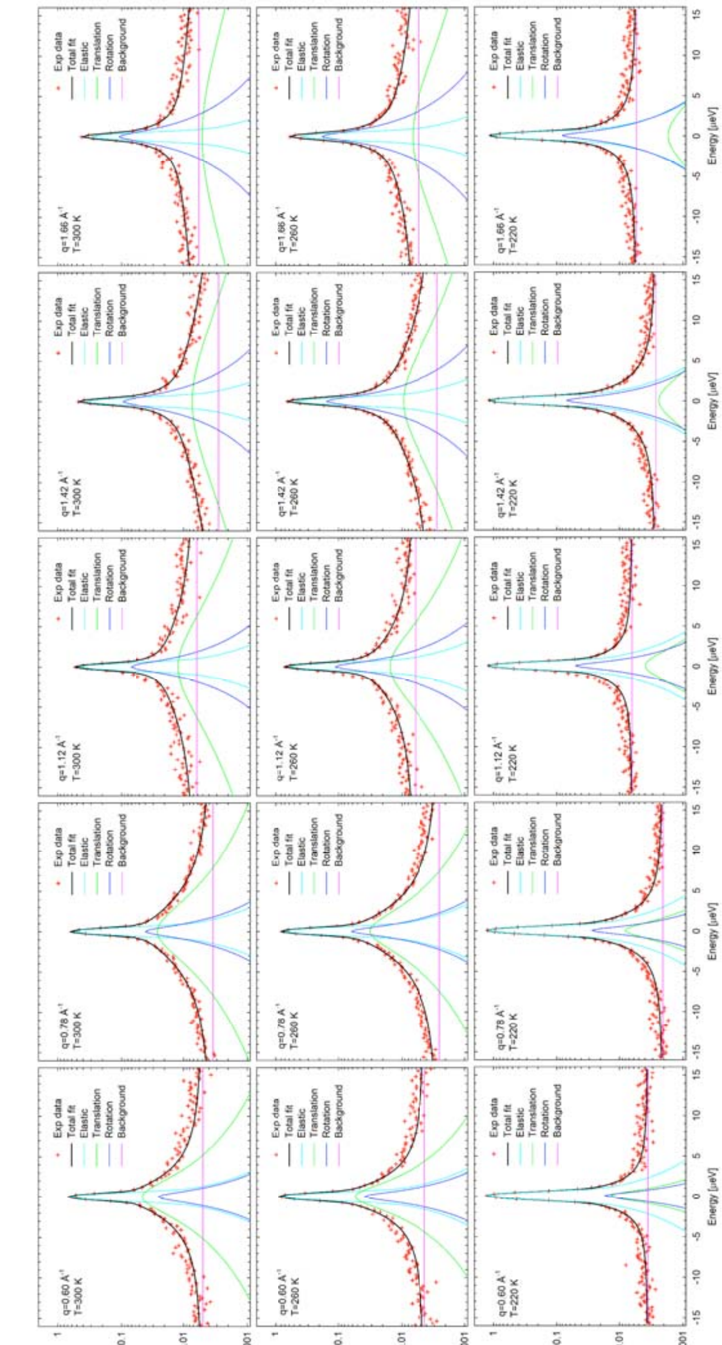

Supplementary Figure 2a: Neutron spectra and fits resulting from a model in which water molecules either translate, rotate or remain immobile. Quasi-elastic neutron scattering spectra of hydration water on the surface of D-tau-H_2O at 220, 260 and 300 K and for five different q-values. The continuous lines represent the fitting curves (see main text). Reduced χ^2 at 220, 260 and 300 K are 2.1, 1.7 and 1.6, respectively.

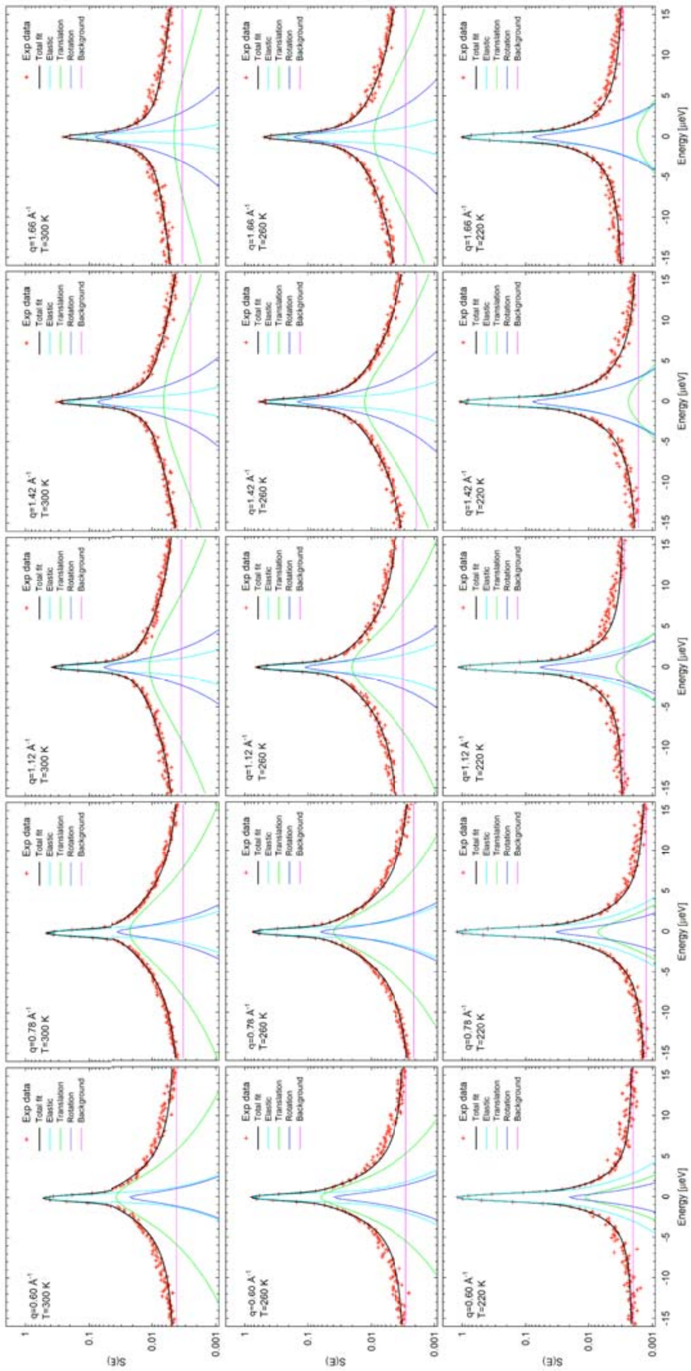

Supplementary Figure 2b: Neutron spectra and fits resulting from a model in which water molecules either translate, rotate or remain immobile. Quasi-elastic neutron scattering spectra of hydration water on the surface of D-MBP-H_2O at 220, 260 and 300 K and for five different q-values. The continuous lines represent the fitting curves (see main text). Reduced χ^2 at 220, 260 and 300 K are 1.6, 1.4 and 1.4, respectively.

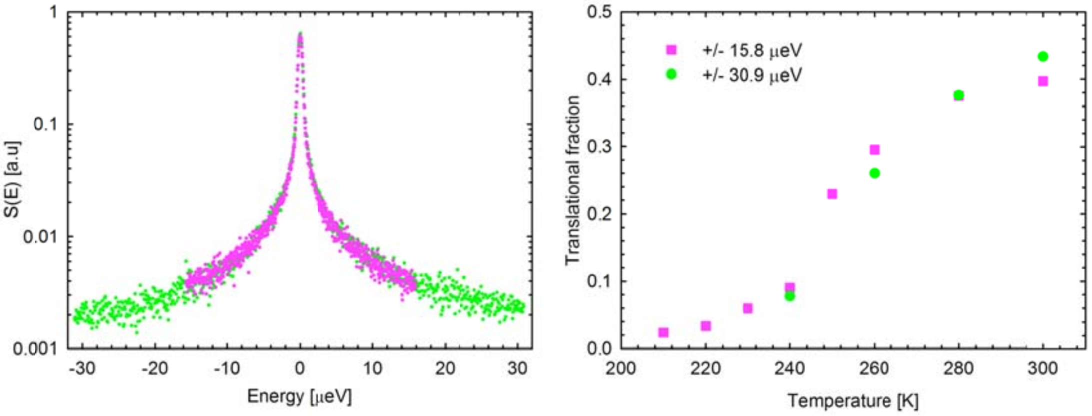

Supplementary Figure 3: **Effect of the energy range on data analysis.** Left panel: comparison of QENS spectra of D-tau-H_2O at 280 K, q=0.78 Å^{-1}, collected using two different energy ranges, +/- 30.9 μeV (green points) and +/- 15.8 μeV (magenta points). Right panel: fraction of the translational contribution as a function of temperature, relative to the analyses of D-tau-H_2O QENS data in the reduced (magenta squares) and extended (green circles) energy ranges.

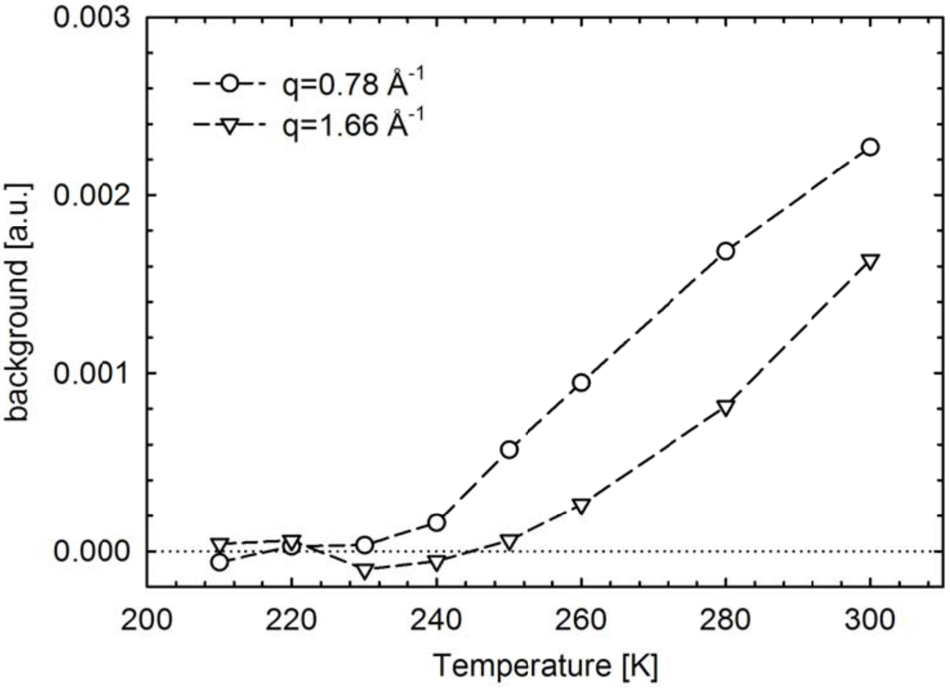

Supplementary Figure 4: Temperature dependence of the flat background obtained by the analysis of D-tau-H_2O data.

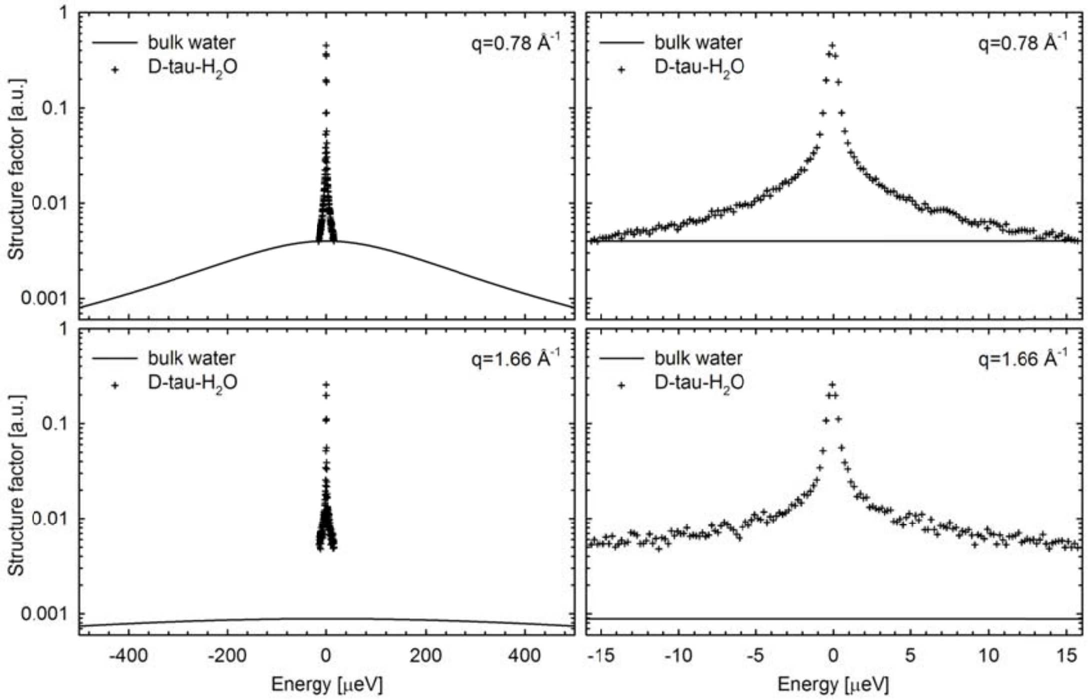

Supplementary Figure 5: A fast translational term is revealed on SPHERES as a flat background. D-tau-H_2O QENS spectra at T=300 K for two different *q* values: *q*=0.78 Å^{-1} (top panels) and *q*=1.66 Å^{-1} (bottom panels), superimposed on a water translational contribution calculated using a typical diffusion coefficient of bulk water (i.e. D=1×10^{-5} cm^{2}s^{-1}). The right panels are the same of the left plots zoomed in the SPHERES energy window.

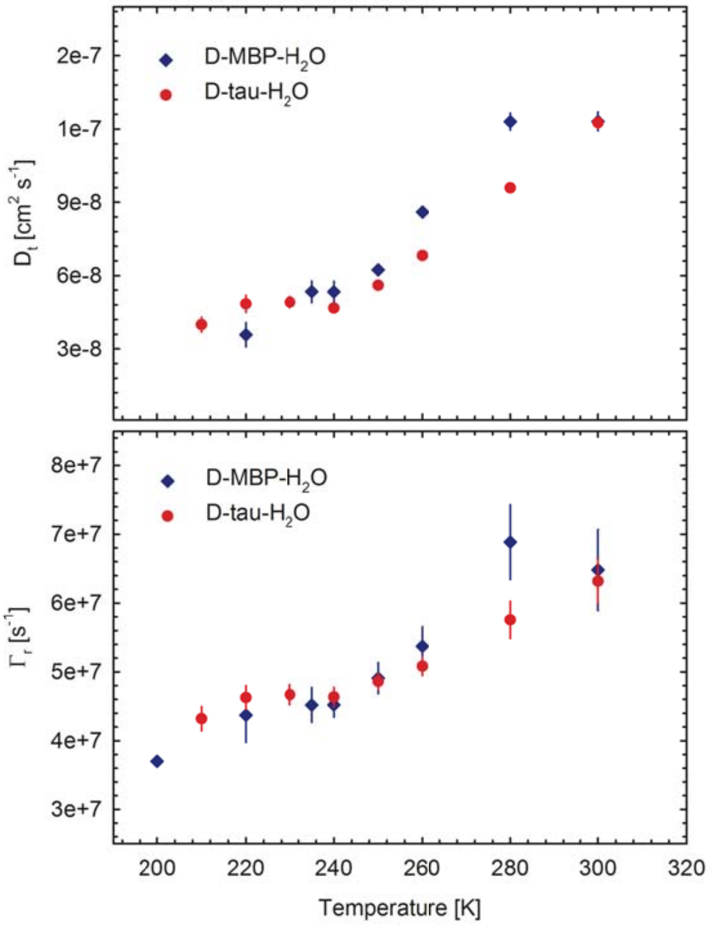

Supplementary Figure 6: Translational diffusion coefficients and rotational correlation rates of hydration water. Temperature dependence of the translational diffusion coefficients (top panel), and rotational correlation rates (bottom panel) obtained by the analysis of D-MBP-H_2O and D-tau-H_2O data.

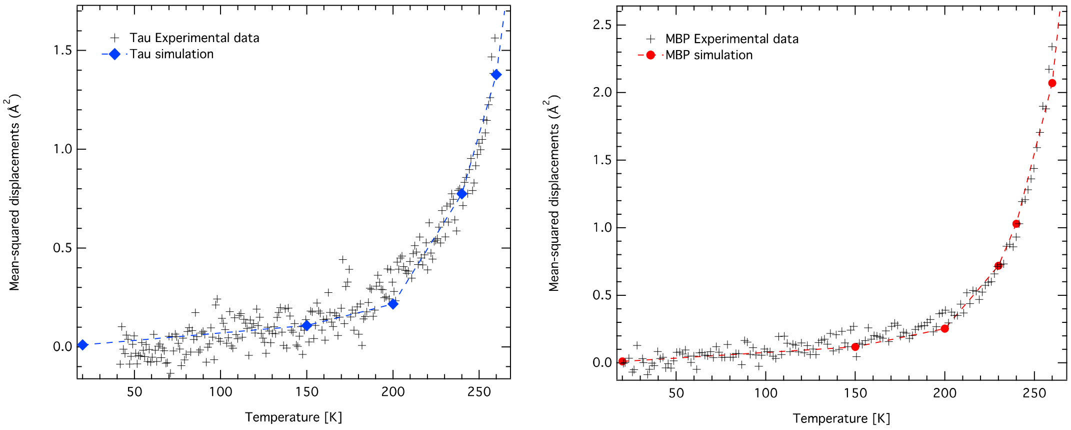

Supplementary Figure 7: Comparing MSD from neutron scattering and MD simulations. Mean-squared displacements of tau (left panel) and MBP (right panel) hydration water measured by elastic incoherent neutron scattering (black crosses; data are extracted from Gallat et al.[5] and Wood *et al.*[6]) and computed from the MD trajectories at 100 ps. The simulated MSD are scaled to the experimental values at 240 K.

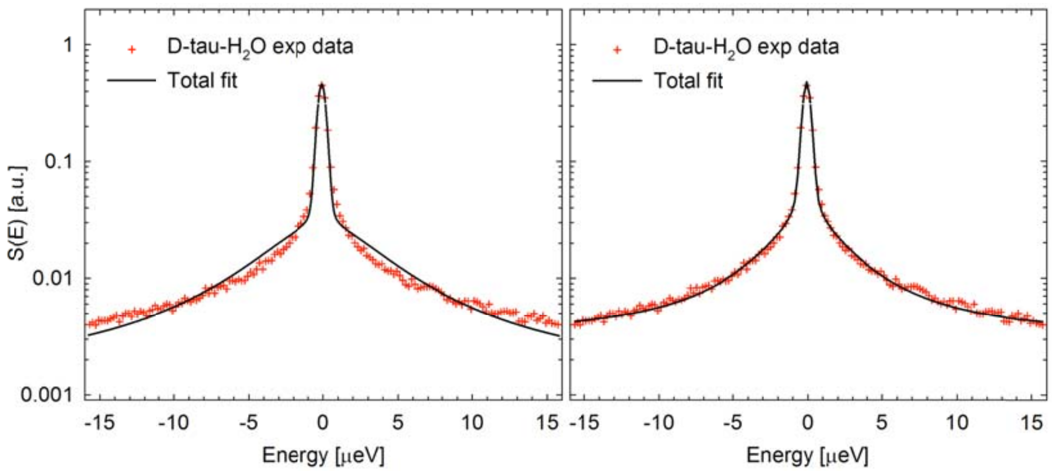

Supplementary Figure 8: Convoluting versus adding rotational and translational components. Results of fitting procedures using a convolution between translational and rotational contributions (left panel) and the model used in the present work, where the two contributions are summed (right panel). Data refers to the D-tau-H_2O sample at 300 K, q=0.78 Å^{-1}.

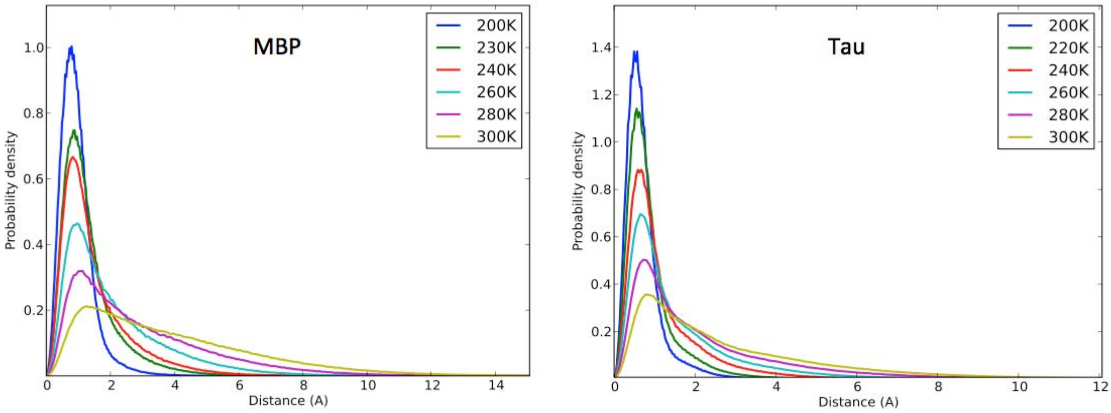

Supplementary Figure 9: Water displacement probability densities ($4\pi r^2G(r,t)$). They are computed from MD simulations of MBP (left panel) and tau (right panel) powder models at t = 100 ps.

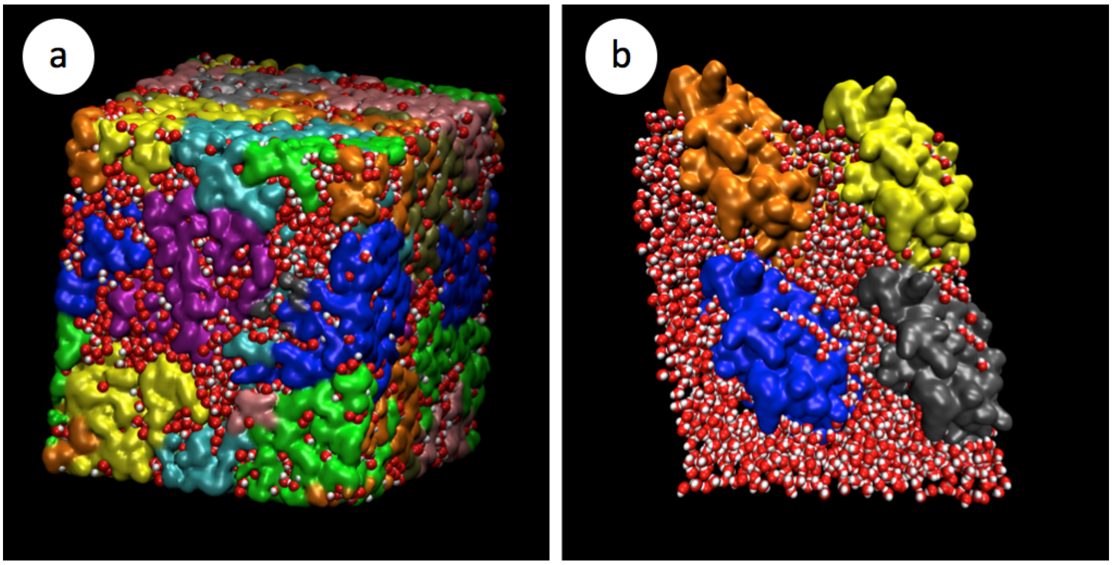

Supplementary Figure 10: Snapshots of the simulation boxes of the tau (a) and MBP (b) powder models. The protein molecules are drawn in surface representation, with different colors for each individual molecule. Water molecules are drawn with van der Waals spheres, with O atoms red and H atoms white.

Supplementary Discussion

Effect of the energy range on data analysis. In order to test a possible effect of the limited energy range on the data analysis, and in particular the influence of the background determination on the fitting parameters, we collected on SPHERES a set of QENS spectra of D-tau-H_2O at four selected temperatures (240, 260, 280, and 300 K) in the energy range +/-30.9 μeV. As evidenced by the plots in Supplementary Figure 3 (left panel), the spectra are compatible with the data collected in the +/-15.8 μeV range, while a data analysis with the same model described above produces results perfectly in agreement with what we obtained in the reduced energy range. As a representative result, in Supplementary Figure 3 (right panel) we show a comparison between the temperature dependence of the translational fraction obtained in the two energy ranges, which confirms that the main results of our analysis are not affected by the choice of the energy range.

On the temperature dependence and physical origin of the background. In Supplementary Figure 4 we plot the constant background $k(q)$, obtained from D-tau-H_2O data analysis (eq. 1 in main text), as a function of temperature, at two different q values, *i.e.*, 0.78 and 1.66 $Å^{-1}$. The data points are vertically offset by subtracting the mean value of the points at temperatures below 240 K, in order to eliminate contributions originating from the instrumental q dependent background and from possible dynamic terms independent of (or barely dependent on) temperature. It is evident that a transition at about 240 K is present at both q values, yet the absolute value is larger at smaller q. The same trend (i.e. $k(q)$ decreasing with the increasing q) is observed at all q values. The observed change as a function of temperature reveals that a dynamical component is contained in the background outside of the instrumental contribution. What is the origin of this dynamical component? Since the quasi-elastic broadening in our data is essentially due to hydration water dynamics, the origin has to be due to water motions with a relaxation rate faster than the time window probed by SPHERES. As discussed in the main text, the rotational and translational components are characterized by different behaviors as a function of q. In particular, the rotational term has a width independent of q and an intensity that increases with the increasing q. A contribution with these characteristics would be detected by SPHERES as an increase of the background at high q. As a consequence, it is not compatible with the observed q dependence of $k(q)$ (Supplementary Figure 4). On the other hand, the translational term has an intensity independent of q and a width that increases with increasing q. Such a contribution would be detected by SPHERES as a reduction of the background at high q, which is compatible with the observed q dependence of $k(q)$.

The plots in Supplementary Figure 5 show how a fast translational term (compared to the SPHERES time window) is revealed by SPHERES as a flat background that decreases at high q. We calculated the line shape of a bulk water component (continuous line in Supplementary Figure 5), using a typical value for the room temperature diffusion coefficient (i.e. $D=1\times10^{-5}$ cm^2s^{-1}), and superimposed it to the experimental spectra collected at SPHERES (crosses in Supplementary Figure 5), at two different q values, i.e. 0.78 and 1.66 $Å^{-1}$. The intensity of the bulk-like contribution is set to an arbitrary value for clarity. The difference in the shape of the calculated translational term between q=0.78 $Å^{-1}$ and q=1.66 $Å^{-1}$ (Supplementary Figure 5 left panels) is due to the increase of

its width Γ according to the relation $\Gamma = D \times q^2$ for Brownian diffusion. When zooming in the SPHERES energy window, the broadening of the calculated translational term is detected as a decrease of the background intensity (Supplementary Figure 5 right panels), in agreement with our experimental observation (Supplementary Figure 4).

Dynamical parameters and comparison with the literature. We report in Suppleme,ntary Figure 6 the temperature dependence of translational diffusion coefficient and rotational correlation rate of hydration water, obtained by the analysis of D-MBP-H_2O and D-tau-H_2O data. The translational diffusion coefficient shows that hydration water has a greatly reduced water mobility with respect to bulk water: the value at room temperature is about two orders of magnitude smaller than the corresponding bulk water value (1.2×10^{-7} cm^2s^{-1} for protein hydration water *vs.* 10^{-5} cm^2s^{-1} for bulk water). The same effect has been recently observed by M. Rosenstihl and M. Vogel using nuclear magnetic resonance on myoglobin and lysozyme powders hydrated at a similar hydration level (h=0.3). They found that in the temperature range 230 - 300 K, water diffusion is a factor of 30 - 100 slower than in bulk water[1]. Dielectric spectroscopy on hydrated protein powders is mainly sensitive to rotational relaxation of hydration water, due to the large permanent dipole moment of the water molecule. The time scale of the rotational component detected in our work is compatible with the relaxation time detected by dielectric spectroscopy on hydrated protein powders and usually attributed to hydration water dynamics (see e.g. [2-4]).

Comparison of water mean-squared displacements obtained from MD simulations with those extracted from elastic incoherent neutron scattering data. We computed the water hydrogen and exchangeable protein hydrogen mean-squared displacements (MSD) at different temperatures and extracted their values at 100 ps. The values of the MSDs at t = 100 ps showed a very similar temperature dependence to water MSDs determined by analysis of elastic incoherent neutron scattering data obtained previously on powders of D-MBP-H_2O and D-tau-H_2O powders.[5] However, the magnitudes of the MSDs at t = 100 ps were roughly an order of magnitude greater than the experimental values. The discrepancy is largely due to the differences in the way the MSDs are determined from simulations and neutron scattering experiments. The simulation values are computed directly from space-time trajectories, while the neutron values are obtained by fitting the q-dependence of the elastic intensity within a Gaussian approximation over a restricted range of q. Both quantities are measures of water mobility, but they cannot be directly compared. To enable a closer comparison of the temperature dependence of the MSDs, we scaled the values computed from the simulation trajectories at t = 100 ps to match the experimental value at 240 K. As can be seen in Supplementary Figure 7, the agreement between the scaled simulation values and the experimentally derived MSDs is very good for both proteins.

Accuracy of fitting with a sum or a convolution of rotational and translational water diffusion. The choice of the model function (see eq. 1 in the main text) used to fit the data is the result of a thorough exploration of different model functions based on the rotational-translational nature of water motions. First, we tried to fit the data with a single rotational-translational function where translational and rotational terms were convoluted

(representing a single homogeneous population of water molecules). The statistical accuracy obtained was insufficient to justify using the model (Supplementary Figure 8 left panel), indicating the presence of more than one population of water molecules. Then we added a second rotational-translational term: in this case the model fitted the experimental data well but the number of independent parameters was too high to extract a meaningful trend in the fitting parameters. Finally, the choice of the sum of a translational and a rotational term (plus the other terms described in the main text) matched both the requirements of a high accuracy of fitting results (Supplementary Figure 8 right panel) and a small number of meaningful free parameters. The physical basis of such a model in terms of dynamical heterogeneity and finite instrumental dynamical window is discussed in the main text. Briefly, the only scenario implying a single rotational-translational term is that, in the time window probed by SPHERES, a homogeneous contribution is observed, arising from one population of water molecules with the same dynamical properties. This is in disagreement with several experimental evidences about the heterogeneity of water populations and water motions on the protein surface. The finding that a model with two decoupled contributions is necessary to fit the quasi-elastic spectra of protein hydration water within the time window probed by SPHERES, reveals the presence of two contributions from distinct (or barely overlapping) water populations: one very slow and predominantly rotational (probably from hindered molecules strongly interacting with protein surface or in docking sites) and the other one predominantly translational (probably from more free water molecules) whose rotational term is larger than the SPHERES energy window.

Water displacement distributions from MD simulations reveal the existence of two populations of hydration water molecules with different dynamics. The global fitting procedure used to analyze the neutron scattering data employed a model in which it was assumed that there are distinct populations of water molecules undergoing primarily librational/rotational or translational dynamics. To assess the validity of this assumption, we used the MD simulation trajectories to calculate displacement distribution functions, $4\pi r^2 G(r,t)$, for the H atoms of the water molecules. $G(r,t)$ is the van Hove correlation function, the time and space Fourier transform of which is the dynamic structure factor probed by neutron spectroscopy, and $4\pi r^2 G(r,t)dr$ is the probability that a tagged particle at $t = 0$ has been displaced by a distance r at time t. The displacement distribution function, $4\pi r^2 G(r,t)$, is the corresponding probability density. The displacement distribution functions for protein/membrane hydration water are qualitatively different from those of bulk water[7]. As expected for Brownian particles, in bulk water under ambient conditions the displacement distribution is a unimodal function whose mean moves to larger distances r and width increases with increasing time t. In protein/membrane hydration water, the displacement distributions exhibit the existence of two classes of water molecules, the populations of which change with temperature, as exemplified by the distributions for water H atoms plotted in Supplementary Figure 9 for MBP and tau hydration water in model powders at $t = 100$ ps. At 200 K, the distributions are dominated by a sharp peak at ~1 Å, which corresponds to water molecules librating and rotating but translationally confined to a solvation cage consisting of surrounding water and protein atoms. As the temperature is increased, the peak corresponding to translationally retarded, librating/rotating water molecules shrinks, while a shoulder,

corresponding to water molecules escaping their solvation cages *via* translational diffusion, grows and extends to larger r.

Supplementary References

1 Rosenstihl, M. & Vogel, M. Static and pulsed field gradient nuclear magnetic resonance studies of water diffusion in protein matrices. *J Chem Phys* 135, 164503 (2011).

2 Khodadadi, S. *et al.* The origin of the dynamic transition in proteins. *J Chem Phys* 128, 195106 (2008).

3 Schiro, G., Cupane, A., Vitrano, E. & Bruni, F. Dielectric relaxations in confined hydrated myoglobin. *J Phys Chem B* 113, 9606-9613, (2009).

4 Jansson, H., Bergman, R. & Swenson, J. Relation between solvent and protein dynamics as studied by dielectric spectroscopy. *J Phys Chem B* 109, 24134-24141, (2005).

5 Gallat, F.-X. *et al.* Dynamical coupling of intrinsically disordered proteins and their hydration water: comparison with folded soluble and membrane proteins. *Biophys J* 103, 129-136 (2012).

6 Wood, K. *et al.* Coincidence of dynamical transitions in a soluble protein and its hydration water: direct measurements by neutron scattering and MD simulations. *Journal of the American Chemical Society* 130, 4586-4587, (2008).

7 Tobias, D. J., Sengupta, N. & Tarek, M. Hydration dynamics of purple membranes. *Faraday Discuss* 141, 99-116 (2009).
